# Neuroprotective Benefits of Exercise and MitoQ on Memory Function, Mitochondrial Dynamics, Oxidative Stress, and Neuroinflammation in D-Galactose-Induced Aging Rats

**DOI:** 10.3390/brainsci11020164

**Published:** 2021-01-27

**Authors:** Jae-Hoon Jeong, Jung-Hoon Koo, Jang Soo Yook, Joon-Yong Cho, Eun-Bum Kang

**Affiliations:** 1 Department of Physical Education, Hanyang University, 222, Wangsimni-ro, Seongdong-gu, Seoul 04763, Korea; trainerhoon@gmail.com; 2 Exercise Biochemistry Laboratory, Korea National Sport University, 1239, Yangjae-daero, Songpa-gu, Seoul 05541, Korea; mt634@knsu.ac.kr (J.-H.K.); chojy86@knsu.ac.kr (J.-Y.C.); 3 Center for Functional Connectomics, Brain Research Institute, Korea Institute of Science and Technology, Hwarang-ro 14-gil 5, Seongbuk-gu, Seoul 02792, Korea; yookjs@kist.re.kr; 4 Division of Sports Science, College of Health and Medical Science, Daejeon University, 62 Daehak-ro, Dong-gu, Daejeon 34520, Korea

**Keywords:** aging, treadmill exercise, MitoQ, NADPH oxidase, mitochondrial dynamics, neuroinflammation

## Abstract

Exercise and antioxidants have health benefits that improve cognitive impairment and may act synergistically. In this study, we examined the effects of treadmill exercise (TE) and mitochondria-targeted antioxidant mitoquinone (MitoQ), individually or combined, on learning and memory, mitochondrial dynamics, NADPH oxidase activity, and neuroinflammation and antioxidant activity in the hippocampus of D-galactose-induced aging rats. TE alone and TE combined with MitoQ in aging rats reduced mitochondrial fission factors (Drp1, Fis1) and increased mitochondrial fusion factors (Mfn1, Mfn2, Opa1). These groups also exhibited improved NADPH oxidase activity and antioxidant activity (SOD-2, catalase). TE or MitoQ alone decreased neuroinflammatory response (COX-2, TNF-α), but the suppression was greater with their combination. In addition, aging-increased neuroinflammation in the dentate gyrus was decreased in TE but not MitoQ treatment. Learning and memory tests showed that, contrarily, MitoQ alone demonstrated some similar effects to TE but not a definitive improvement. In conclusion, this study demonstrated that MitoQ exerted some positive effects on aging when used as an isolated treatment, but TE had a more effective role on cognitive impairment, oxidative stress, inflammation, and mitochondria dysfunction. Our findings suggest that the combination of TE and MitoQ exerted no synergistic effects and indicated regular exercise should be the first priority in neuroprotection of age-related cognitive decline.

## 1. Introduction

Aging is an inevitable process of physical and functional decline, including a gradual decrease in cognitive ability, such as reduced memory and spatial ability [1]. Age-related changes in the brain increase vulnerability to various diseases and ultimately result in a lower quality of life. Successful aging generally refers to the absence of disease or impairment, preservation of physical and cognitive function, and continued participation in social and productive activities [2]. Maintaining one’s dignity and value as a human being is crucial, even during the aging process.

Among several identified lifestyle changes for preventing cognitive decline with aging, recent attention has turned towards a non-pharmacological treatment, such as physical activity and dietary supplements, due to the lack of pharmacological effects and the rising cost of conventional medicine. Indeed, a combination of exercise and diet with an antioxidant supplementation, such as epicatechin and astaxanthin, has been shown to induce synergistic effects on the hippocampus-dependent function [3,4]. Contrarily, several studies have revealed that exercise alone, but not in combination with dietary changes, improves learning and memory functions [5,6], raising the question of whether a combined intervention could have greater effects on cognitive function than exercise alone. Although the possible mechanisms underlying exercise and/or antioxidant treatment-enhanced hippocampal function involve neuronal plasticity and its related molecular levels with neurotropic factors [4,7], a specific-mechanism, such as mitochondrial dynamics and neuro-immune system, are not fully understood.

Mitochondria are dynamic intracellular organelles responsible for biological oxidation in most eukaryotic cells, whose dynamics are regulated by repeated processes of fusion (elongation of the mitochondria) and fission (fragmentation of the mitochondria) [8,9]. Several fusion- and fission-related proteins are involved in mitochondrial dynamics, which are essential for maintaining normal mitochondria function [10,11]. However, irregular expression of the proteins can lead to abnormal changes in mitochondria. In particular, mitochondrial dysfunction is a feature of aging [12], and persistent inflammation, oxidative stress, and mitochondrial dysfunction contribute to reduced brain function by accelerating brain aging [13,14]. In addition, aging is accompanied by low level of chronic inflammation in the immune system and central nerve system (CNS), which is known to contribute to many age-related diseases [15,16]. Neuroinflammation in the CNS progresses when glial cells, such as microglia and astrocytes, become persistently activated in response to oxidative stress [17]. Reactive oxygen species (ROS) are generated by NADPH oxidase (NOX), which is a membrane-bound enzyme complex. There are several subtypes of NOX, among which NOX2 is expressed in various cell types, including neurons and endothelial cells, and is expressed at high levels in microglial cells involved in the immune response and inflammation [18,19,20]. NOX2 comprises both membrane (gp91phox, p22phox) and cytosolic (p47phox, p67phox) subunits. Elevated ROS levels due to NOX activity promote inflammation, activating glial cells, which in turn further enhance the secretion of proinflammatory cytokines, oxidative stress, and free radical injury, ultimately leading to neuronal cell death [21,22,23].

Brain aging plays an important role in cognitive impairment and is closely related to neurodegenerative disorders. Although it is impossible to prevent the general decline in brain function that occurs during natural aging, physical activity and the consumption of antioxidants are useful as noninvasive methods to maintain brain function, especially cognitive function. In particular, exercise reportedly ameliorates brain dysfunction by promoting neuroplasticity, improving metabolic efficiency, and increasing tolerance to oxidative stress [24,25]. However, the molecular mechanisms responsible for enhancing cognitive function or preventing its decline remain unknown. In terms of antioxidants, the mitochondrial-targeted antioxidant mitoquinone (MitoQ) is known to cross the inner mitochondrial membrane and accumulate within mitochondria, unlike other antioxidants, to reduce ROS levels generated in mitochondria directly [26]. Although few studies have been conducted on MitoQ, various reports describe its antioxidative effects. Studies by Gioscia-Ryan et al. [27] and Rossman et al. [28] demonstrated that MitoQ reduced ROS levels and increased NO production in mitochondria of aged blood vessels. Vergeade et al. [29] and Braakhuis et al. [30] reported that MitoQ maintained the activities of antioxidant enzymes, such as SOD-2, catalase, and GPx, during the aging process. Regarding brain aging, further research is warranted to elucidate the molecular mechanisms responsible for the positive effects of exercise and MitoQ on declining brain function.

Until now, aging research using several animal models has been investigated the underlying mechanisms of brain aging. In particular, D-galactose (D-gal) administration reportedly causes aging in animals that resembles human aging, including memory loss, neurodegeneration, changes in biochemical markers of oxidative stress, decreased immune activity, and abnormal regulation of gene expression [31]. Rodents administered D-gal show a gradual decline in learning and memory, increased production of free radicals in the brain, impaired calcium homeostasis, and mitochondrial dysfunction [32,33], thus providing an animal model of brain aging [34]. Therefore, using the D-galactose-induced aging rat model, we investigated the effects of treadmill exercise (TE) and MitoQ, as independent or combined treatments, on mitochondrial fission and fusion, inflammation, and antioxidant activity, as well as hippocampus-dependent cognition. We hypothesized that an 8-week combined treadmill exercise and MitoQ in D-galactose-treated rats would either additively or synergistically improve the decline of learning and memory function and induce beneficial changes in the levels of proteins involved in hippocampal neuroprotection.

## 2. Materials and Methods

### 2.1. Experimental Animals

Six-week-old male Sprague-Dawley (SD) rats were obtained from JA Bio (Gyeonggi-do, Korea). The rats were reared in the Korea National Sports University Animal Laboratory (22 ± 2 °C, 50% ± 5% humidity, and 12/12 h light/dark cycle). Food and drinking water were provided ad libitum. The rats were divided into 5 groups: young control group (Y-CON, *n* = 12), D-galactose group (D-CON, *n* = 12), D-galactose plus TE group (D-TE, *n* = 12), D-galactose plus MitoQ group (D-MI, *n* = 12), and D-galactose plus TE and MitoQ group (D-COMBI, *n* = 12). Rats were euthanized after completing 8 weeks of TE and memory behavior tests by CO_2_ inhalation using a euthanasia chamber. The study protocol received approval from the Korea National Sports University Institutional Animal Care and Use Committee (KNSU-IACUC-2018-05).

### 2.2. Drug Administration

The method described by Lei et al. [31] was employed to induce the aging of the experimental animals. Specifically, D-gal (Sigma-Aldrich, St. Louis, MO, USA) was dissolved in normal saline, and a 100 mg/kg dose was administered by intraperitoneal (IP) injection once per week for 10 weeks. The feeding method suggested by Smith and Murphy [26] was adapted for the administration of MitoQ in this experiment. MitoQ was mixed with sterile saline for a final dilution of 0.1 mM/mL. D-galactose-induced aging rats were then injected intraperitoneally with 100 µM/kg MitoQ (twice per week) for 8 weeks.

### 2.3. Treadmill Exercise (TE)

The D-TE and D-COMBI groups were subjected to a progressive loading exercise program using a rodent treadmill (8 Lanes, Daemyung Scientific Co, Ltd., Seoul, Korea). The animals first performed acclimation training for 1 week (2 m/min for the first 5 min, 5 m/min for the next 5 min, and 8 m/min for the last 20 min). The main exercise program was performed 5 days per week over the next 8 weeks. This program was designed as follows, with reference to the progressive exercise program suggested by Hong et al. [35]: 10–12 m/min for 10 min in Week 1; 10–12 m/min for 20 min in Week 2; 18–20 m/min for 20 min in Week 3; 18–20 m/min for 30 min in Week 4; and 18–20 m/min for 50 min in Weeks 5–8. The incline of the treadmill was fixed at 0%.

### 2.4. Passive Avoidance Task

The passive avoidance task is a fear-motivated test used to measure the working memory ability of small laboratory animals. In the present study, the passive avoidance task was performed 3 days before the end of the TE program. The apparatus for the passive avoidance task consisted of two chambers: The front chamber was a well-lit, bright, white box (18 × 18 × 25 cm^3^), connected to the rear chamber, which was a dark black box (18 × 18 × 25 cm^3^). The rear chamber had a stainless-steel floor capable of delivering electric shocks. The wall between the two chambers had a guillotine-style door that could be opened and closed. The time taken for the animal to enter the rear chamber via the front chamber was measured (initial latency time), and then an electric shock (0.5 mA) was delivered for 2 s. The animal was removed after 5 s and returned to its rearing cage. The task was repeated 72 h later using an identical method, and the time for the animal to enter the rear chamber via the front chamber was recorded (retention latency time) up to a maximum of 300 s.

### 2.5. Morris Water Maze Test

The Morris water maze test is used to assess the spatial learning and memory of rodents. The test was performed in a room with controlled temperature (21–23 °C), humidity (50–60%), and lighting using a circular water tank (diameter 120 cm × height 15 cm) filled with water (20–24 °C). Skim milk powder was added to the water to obscure the escape platform (10 cm diameter), which was placed on the floor of the tank approximately 1 cm below the water surface. The distance traveled by the animal before reaching the platform (escape distance) and time taken to reach the platform (escape latency time) were measured before and after the TE program using EthoVision XT8 video tracking software (Noldus, Wageningen, The Netherlands) and a camera installed on the ceiling above the center of the tank. The water tank was divided into quarters labeled Zones 1–4, and the platform was placed in Zone 1. For each treatment group, the water maze test was performed 5 days per week. The rat always started in the same position and was allowed two 60-s practice attempts to try to reach the platform, which was always in the same location. After 48 h of rest, the platform was removed on the seventh day; escape distance and escape latency time were measured for 60 s with the rat starting at the same position.

### 2.6. Tissue Preparation

After completing 8 weeks of TE and memory behavior tests, all rats were euthanized by CO_2_ inhalation, and brain tissues were harvested from 7 animals in each treatment group. After separating the cerebral cortex and the hippocampus, brain tissue was flash-frozen in liquid nitrogen and stored at −80 °C until analysis. The remaining 5 animals were used for immunohistochemistry (IHC) analysis. After opening the thoracic cavity, 50 mM phosphate-buffered saline (PBS) was passed through the left ventricle for 10 min, and then the animal was perfused with 4% paraformaldehyde (PFA) in 100 mM PBS. After perfusion fixation, the brain was harvested, placed in 4% PFA, and fixed for 4 h at 4 °C. The fixed brain tissue was submerged in 30% sucrose solution for 2 days and then cut into 40 µm-thick slices using a cryostat (Leica Microsystems, Nussloch, Germany).

### 2.7. Mitochondria Isolation

To isolate the mitochondria, we used a Mitochondria Extraction Kit (IMGENEX Corporation, San Diego, CA, USA) according to the manufacturer’s instructions. For every 100 mg of hippocampal tissue, 1 mL of homogenizing buffer was added. After homogenization, the tissue was centrifuged at 900× *g* for 10 min at 4 °C, and the supernatant was collected and centrifuged again at 15,000× *g* for 30 min at 4 °C. Taking the centrifuged cytosolic fraction, the cytosol was separated, and the remaining pellet was mixed thoroughly in 1 mL of suspension buffer, before centrifuging at 15,000× *g* for 10 min at 4 °C. The supernatant was removed, and another 1 mL of suspension buffer was added before mixing thoroughly and centrifuging again at 15,000× *g* for 10 min at 4 °C. The supernatant was removed, and the remaining pellet was dissolved in 1 mL of complete mitochondrial lysis buffer for 30 min at 4 °C. The mitochondrial extract was then centrifuged at 15,000× *g* for 5 min at 4 °C, and the supernatant (mitochondrial fraction) was collected.

### 2.8. Western Blotting

The total protein obtained by mitochondrial isolation was quantified using the Bradford method. An equal amount of mitochondrial proteins (30 µg per lane) was loaded onto sodium dodecyl sulphate–polyacrylamide gel electrophoresis (SDS-PAGE) (8% or 12%). After electrophoresis, proteins were transferred to a Polyvinylidene fluoride (PVDF) membrane (Millipore, Boston, MA, USA). Blocking was performed for 1 h at room temperature using 1×Tris-buffered saline containing Tween-20 (TBS-T) solution with 5% BSA, and then the membrane was reacted with primary antibodies overnight at 4 °C. The following primary antibodies were used: Mfn1, Mfn2, Opa1, Drp1, Fis1, p22 phox, p47 phox, gp91 phox, SOD-2, catalase, and β-actin (Santa Cruz Biotechnology, Dallas, TX, USA, dilution: 1:1000); and TNF-α (Abcam, Cambridge, UK, dilution: 1:1000). The membranes were then washed with a washing buffer (PBS with 0.1% Tween 20), after which they were incubated with horseradish peroxidase (HRP)-conjugated goat anti-rabbit secondary antibodies for p22 phox, gp91 phox, and TNF-α (Invitrogen, Carlsbad, CA, USA, dilution: 1:5000). HRP-conjugated goat anti-mouse secondary antibodies were used for Mfn1, Mfn2, Opa1, Drp1, Fis1, p47 phox, SOD-2, catalase, and β-actin (Santa Cruz Biotechnology, dilution: 1:5000). Protein expression levels were detected using the ECL Western blotting detection system (Santa Cruz Biotechnology). Densities of the developed protein bands were analyzed using the ChemiDoc XRS gel imaging system (Bio-Rad Laboratories, Hercules, CA, USA).

### 2.9. Immunohistochemistry (IHC)

For IHC analysis, the free-floating method was used for the selected tissue in each treatment group. After washing the tissue 3× for 10 min each in 0.01 M PBS, the tissue was incubated at 80 °C for 60 min in a beaker containing 0.01 M sodium citrate, followed by blocking in 10% normal donkey serum (Millipore Sigma, Burlington, MA, USA). The tissue sample was then reacted with primary antibodies anti-p22 phox (Santa Cruz Biotechnology, dilution: 1:500) and anti-Glial fibrillary acidic protein (GFAP) (Abcam, dilution: 1:500) for 12 h at 4 °C. The following day, the tissue was washed 3× for 5 min each in 0.01 M PBS and then reacted with HRP-conjugated goat anti-mouse secondary antibody (Santa Cruz Biotechnology, dilution: 1:5000) at room temperature for 2 h. Finally, the tissue was incubated at room temperature using a Vectastain-Elite ABC kit (Vector Laboratories, Burlingame, CA, USA) solution, and the results were visualized using the DAB Peroxidase Substrate Kit (Vector Laboratories). Each tissue slice was mounted on a slide using a mounting medium (Vector Laboratories) and inspected using a light microscope (Leica Microsystems).

### 2.10. Statistical Analyses

Data were analyzed using SPSS for Windows Version 18.0 (SPSS Inc., Chicago, IL, USA). All values are expressed as the mean ± SEM. Statistical significance was determined using one-way ANOVA. Bonferroni post hoc tests were used for multiple comparisons. Differences were considered statistically significant at *p* < 0.05.

## 3. Results

### 3.1. Effects of Treadmill Exercise and MitoQ on the Expression of Mitochondrial Dynamics-Related Proteins in the Hippocampus of D-Gal-Induced Aging Rats

**Figure 1:**
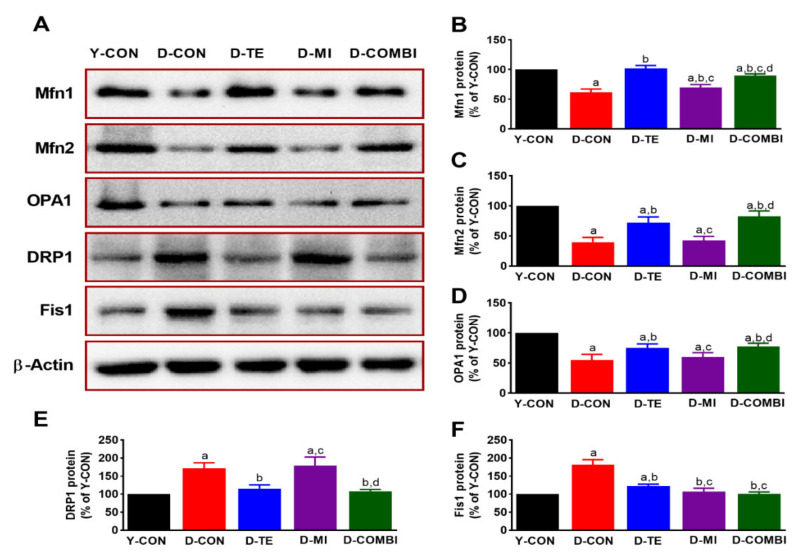
Effect of treadmill exercise and mitoquinone (MitoQ) on the expression of mitochondrial dynamics related proteins in the hippocampus of D-galactose (D-gal) induced aging rats. (**A**) Representative Western blots of mitochondrial fusion- and fission-related proteins. (**B**–**F**) Densitometric analysis of the Western blot bands normalized to β-Actin. The data shown in the Western blot are means from seven rat brains. β-Actin was probed as an internal control. Bonferroni post hoc test was used after ANOVA. Values are means ± SEM. a Denotes statistical difference from the young control group (Y-CON) group. b Denotes statistical difference from the D-galactose group (D-CON) group. c Denotes statistical difference from the D-galactose plus treadmill exercise (D-TE) group. d Denotes statistical difference from the D-galactose plus MitoQ (D-MI) group (*p* < 0.05). D-COMBI; D-galactose plus TE and MitoQ group.

We verified that TE and MitoQ affected mitochondrial fusion-related factors (Mfn1, Mfn2, Opa1) and fission-related factors (Drp1, Fis1) in the hippocampus of aging rats (Figure 1). Expression levels of mitochondrial fusion-related factors differed significantly between treatment groups (Mfn1, F_4,34_ = 134.750, *p* < 0.001; Mfn2, F_4,34_ = 80.816, *p* < 0.001; Opa1, F_4,34_ = 51.456, *p* = 0.001). Expression levels of mitochondrial fission-related factors also differed significantly between treatment groups (Drp1, F_4,34_ = 53.448, *p* < 0.001; Fis1, F_4,34_ = 116.313, *p* < 0.001). The post hoc test results are shown in Figure 1. Compared with the Y-CON group, expression of mitochondrial fusion-related factors decreased in the D-CON group and showed increasing trends in the D-TE, D-MI, and D-COMBI groups. On the other hand, expression of mitochondrial fission-related factor Drp1 increased in the D-CON group compared with the Y-CON group. While mitochondrial fission signals were decreased by combination treatment with TE, MitoQ treatment alone did not induce a significant change. The other mitochondrial fission-related factor, Fis1, demonstrated the same decreasing trends as Drp1 but was also decreased by MitoQ treatment alone.

### 3.2. Effects of Treadmill Exercise and MitoQ on the Expression of NADPH Oxidase Subunits in the Cerebral Cortex and Hippocampus of D-Gal-Induced Aging Rats

**Figure 2:**
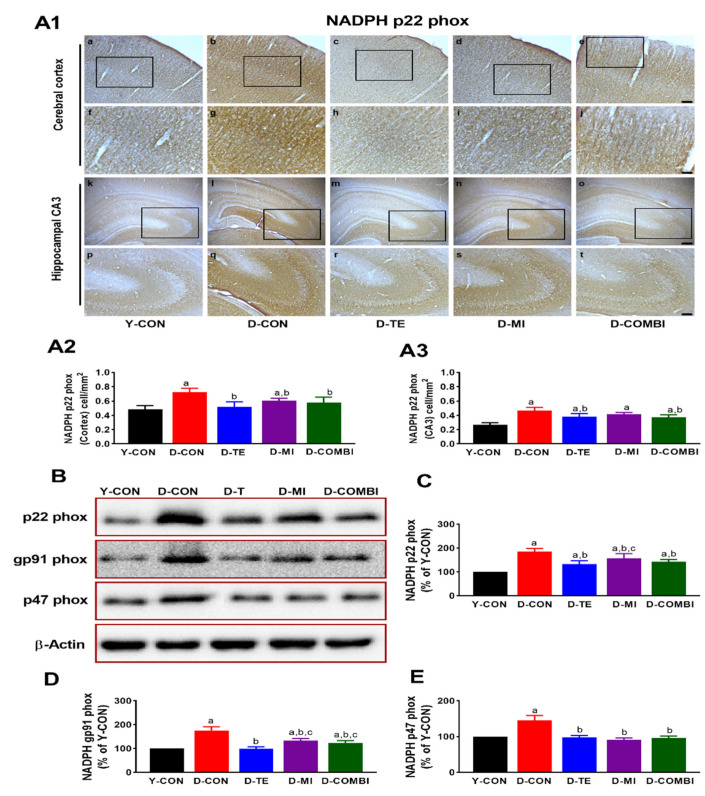
Effects of treadmill exercise and MitoQ on the expression of NADPH oxidase subunits in the cerebral cortex and hippocampus of D-gal induced aging rats. (**A1**) Photomicrographs showing NAPDH p22 phox immunoreactivity in the cerebral cortex and hippocampus of D-gal induced aging rats in each group. Black rectangles in **A1** indicate the portion of the images as shown in **f**–**i** and **p**–**t**. (**A2**,**A3**) Optical density quantification of NADPH p22 phox staining in the cerebral cortex and hippocampus. Bonferroni post hoc test after ANOVA. Values are presented as means ± SEM. (*n* = 5 animals). (**a**–**e** and **k**–**o**) Scale bar = 100 μm, (**f**–**j** and **p**–**t**) Scale bar = 50 μm. (**B**) Representative Western blot images for NADPH oxidase subunits in the hippocampus. (**C**–**E**) Quantitation of NADPH oxidase subunits in the hippocampus. The data shown in the Western blot were means from seven rat brains. β-Actin was probed as an internal control. Bonferroni post hoc test was used after ANOVA. Values are means ± SEM. a Denotes statistical difference from the Y-CON group. b Denotes statistical difference from the D-CON group. c Denotes statistical difference from the D-TE group. (*p* < 0.05).

We observed significant differences between treatment groups in the immunoreactivity of p22 phox in the cerebral cortex and hippocampus of aging rats (Figure 2) (CA3, F_4,24_ = 18.786, *p* < 0.001; cortex, F_4,24_ = 12.662, *p* < 0.001). In the cortex, the D-CON group showed increased immunoreactivity of p22 phox compared with the Y-CON group, which significantly decreased in the D-TE, D-MI, and D-COMBI groups. In CA3, increased immunoreactivity of p22 phox was decreased in the D-TE and D-COMBI groups compared to D-CON. The protein expression levels of NOX subunits p22 phox, gp91 phox, and p47 phox differed significantly between groups (p22 phox, F_4,34_ = 40.513, *p* < 0.001; gp91 phox, F_4,34_ = 57.282, *p* < 0.001; p47 phox, F_4,34_ = 69.249, *p* < 0.001). The results of the post hoc test showed a significant increase in NOX subunit levels in the D-CON compared to the Y-CON. However, NOX subunit levels were lower in all intervention groups compared to the D-CON group. In particular, the expression of p22 phox and gq91 phox was significantly decreased in the D-TE compared to the D-MI group. These findings imply that exercise and MitoQ individually prevent aging-induced oxidative stress in the brain, but their combination results in no additional effects.

### 3.3. Effect of Treadmill Exercise and MitoQ on GFAP Expression in the Cerebral Cortex and Hippocampal Dentate Gyrus of D-Gal-Induced Aging Rats

**Figure 3:**
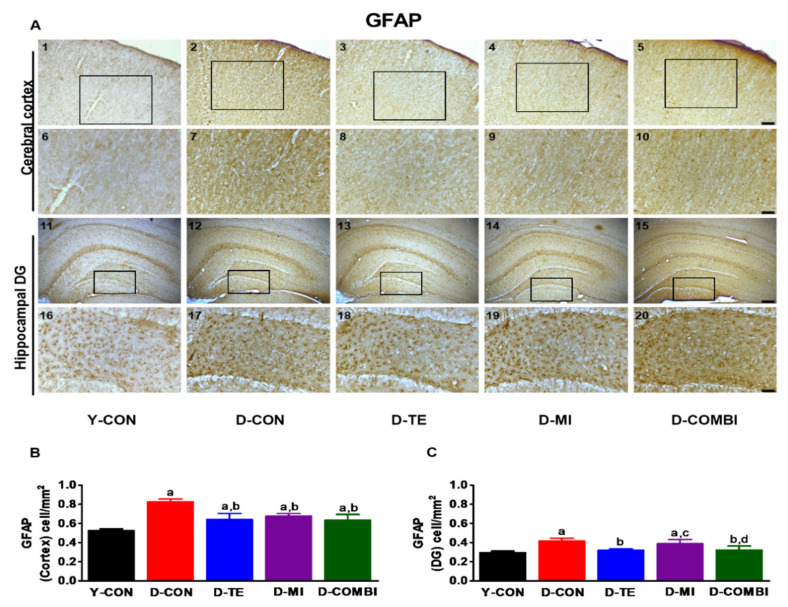
Effects of treadmill exercise and MitoQ on the expression of Glial fibrillary acidic protein (GFAP) in the hippocampal dentate gyrus and cerebral cortex of D-galactose-induced aging rats. (**A**, **1**–**10**) Photomicrographs showing GFAP immunoreactivity in the cerebral cortex of D-galactose-induced aging rats in each group. (**A**, **11**–**20**) Photomicrographs showing GFAP immunoreactivity in the hippocampal dentate gyrus of D-galactose-induced aging rats in each group. (**A**, **6**–**10** and **A**, **16**–**20**) An enlarged view of the area indicated by the black rectangle in A. (**A**, **1**–**5** and **A**, **11**–**15**) Scale bar = 100 μm, (**A**, **6**–**10** and **A**, **16**–**20**) Scale bar = 50 μm. (**B**) Optical density quantification of GFAP staining in the cerebral cortex. (**C**) Optical density quantification of GFAP staining in the hippocampal dentate gyrus. Bonferroni post hoc test after ANOVA. Values are presented as means ± SD. (*n* = 5 animals). a Denotes statistical difference from the Y-CON group. b Denotes statistical difference from the D-CON group. c Denotes statistical difference from the D-TE group. d Denotes statistical difference from the D-MI group (*p* < 0.05).

We observed significant differences between treatment groups in GFAP immunoreactivity in the cerebral cortex and hippocampal dentate gyrus (DG) of aging rats (Figure 3) (Cortex, F4_,24_ = 13.524, *p* < 0.001; DG, F_4,24_ = 30.364). The results of the post hoc test showed that D-gal treatment significantly increased GFAP immunoreactivity in both cortex and DG. Compared to the D-CON group, GFAP levels in the cortex were decreased in all intervention groups, and GFAP levels in DG were decreased in the D-TE and D-COMBI groups, whereas the D-MI group showed differences in GFAP immunoreactivity only in the cortex region. Thus, although TE or MitoQ treatment reduced D-gal-induced neuroinflammation in the cortex, only TE intervention produced positive effects in DG.

### 3.4. Effect of Treadmill Exercise and MitoQ on the Expression of Inflammatory Proteins and Antioxidant Enzymes in the Hippocampus of D-Gal-Induced Aging Rats

**Figure 4:**
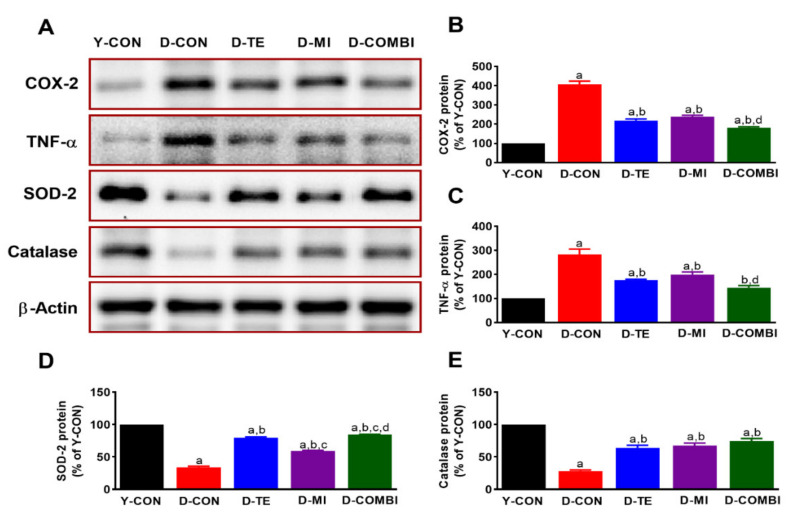
Effect of treadmill exercise and MitoQ on the expression of inflammatory proteins and antioxidant enzymes in the hippocampus of D-gal induced aging rats. (**A**) Representative Western blots of inflammatory proteins (COX-2, TNF-α) and antioxidant enzymes (SOD-2, Catalase). (**B**–**E**) Densitometric analysis of the Western blot bands normalized to β-Actin. The data shown in the Western blot are means from seven rat brains. β-Actin was probed as an internal control. Bonferroni post hoc test was used after ANOVA. Values are means ± SEM. a Denotes statistical difference from the Y-CON group. b Denotes statistical difference from the D-CON group. c Denotes statistical difference from the D-TE group. d Denotes statistical difference from the D-MI group (*p* < 0.05).

TE and MitoQ were confirmed to affect inflammatory proteins (COX-2, TNF-α) and antioxidant enzymes (SOD-2, catalase) in the hippocampus of aging rats (Figure 4). Expression levels of inflammatory proteins differed significantly between treatment groups (COX-2, F_4,34_ = 148.171, *p* < 0.001; TNF-α, F_4,34_ = 33.645, *p* < 0.001). Expression levels of antioxidant enzymes also differed significantly between treatment groups (SOD-2, F_4,34_ = 477.940, *p* < 0.001; catalase, F_4,34_ = 75.447, *p* < 0.001). The results of the post hoc test showed that there was a dramatic change in COX-2, TNF-α, SOD-2, and catalase expression between Y-CON and D-CON groups. However, TE and MitoQ alone reduced D-gal-upregulated COX-2 and TNF-α protein expression, and the combination of TE and MitoQ further mitigated both inflammatory cytokines compared to MitoQ alone. These results suggest that D-gal administration induces neuroinflammation, while TE and MitoQ have anti-inflammatory effects in the hippocampus. In addition, TE and MitoQ alone increased D-gal-induced reduction of SOD-2 and catalase protein expression. SOD-2 was further increased in the D-COMBI group compared to MitoQ alone and TE alone treatment. These results suggest that D-gal administration also induces ROS in the hippocampus, while TE and MitoQ treatment restores the age-induced oxidative stress.

### 3.5. Effect of Treadmill Exercise and MitoQ on Learning and Memory in D-Gal-Induced Aging Rats

**Figure 5:**
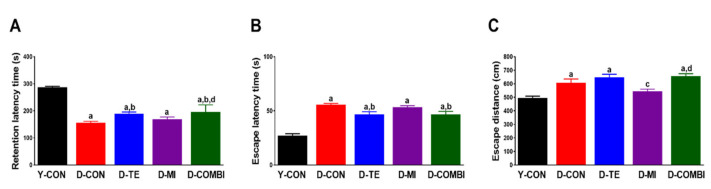
Effect of treadmill exercise and MitoQ on learning and memory tasks in D-gal induced aging rats. (**A**) The working memory test after performing the passive avoidance task 3 days before the end of treadmill exercise (TE). Patterns of (**B**) escape latency time and (**C**) escape distance to cross to the former platform location in the water maze test. Bonferroni post hoc test was used after ANOVA. Values are means ± SEM. a Denotes statistical difference from the Y-CON group. b Denotes statistical difference from the D-CON group. c Denotes statistical difference from the D-TE group. d Denotes statistical difference from the D-MI group (*p* < 0.05).

To determine the functional improvements in TE and MitoQ treatment in the D-gal-induced aging hippocampus, we performed the Morris water maze and passive avoidance tests to assess learning and memory (Figure 5). The passive avoidance test demonstrated significant differences between treatment groups (retention latency time, F_4,59_ = 69.131, *p* < 0.001; Figure 5A). The post hoc test showed that a significant decrease in the retention latency time was observed in the D-CON group compared to the Y-CON group. However, there was a significant increase in retention latency time in the D-TE, but not in the D-MI group, compared to the D-CON group. In addition to the results of Morris water maze test (Figure 5B,C), escape latency times and escape distances differed significantly between treatment groups (escape latency time, F_4,59_ = 31.565, *p* < 0.001; escape distance, F_4,59_ = 10.833, *p* < 0.001). The post hoc test showed that there was a significant increase in escape latency times and distances in the D-CON compared to the Y-CON group. Escape latency time significantly increased in the D-TE and the D-COMBI groups compared to the D-CON group. However, there was no significance of escape distance in all intervention groups compared to the D-CON group. These results suggest that the TE alone restores D-gal-induced impairment of learning and memory function.

## 4. Discussion

Age-associated cognitive decline is closely related to brain aging. There is enormous value in delaying brain aging, although it is an inevitable part of the aging process. In this study, we investigated the preventative effects of TE and MitoQ on cognitive impairment associated with brain aging. D-gal was found to partially impair mitochondrial dynamics by increasing and decreasing mitochondrial fission and fusion in the hippocampus, respectively. In addition, D-gal increased inflammation and NOX expression, resulting in decreased hippocampus-dependent cognitive function related to learning and memory. However, TE effectively restored these pathological conditions and prevented cognitive decline. MitoQ treatment alone ameliorated the effects of aging on inflammation and antioxidant enzymes but did not induce significant changes in mitochondrial dynamics or cognitive function. Regardless of the synergistic or additive effects to brain aging-related outcome measures, our results suggest that TE or MitoQ have potential roles as non-pharmacological treatments for preventing or slowing cognitive decline with aging.

Imbalanced mitochondrial dynamics are closely related to various aging-related diseases and are highly important in cell survival [36,37]. In this study, the D-CON group treated with D-gal to induce aging exhibited increased expression of mitochondrial fission-related proteins Drp1 and Fis1 and decreased expression of mitochondrial fusion-related proteins Mfn1, Mfn2, and Opa1. These results indicate that excessive mitochondrial fission led to a partial reduction in mitochondrial function. On the other hand, TE decreased the expression of the mitochondrial fission-related proteins and increased the expression of mitochondrial fusion-related proteins, helping maintain mitochondrial homeostasis. In the result of MitoQ, only the fusion protein Mfn1 and the fission protein Fis1 showed similar trends to TE, but the expression of the other proteins (Mfn2, Opa1, and Drp1) did not differ from that in the D-CON group. Previous studies using cell culture systems demonstrated that MitoQ improved oxidative stress-disrupted mitochondrial dynamics balance [38], as well as mitochondrial fission in a pharmacological model of Parkinson’s disease, inducing mitochondrial fragmentation [39], which is inconsistent with our findings from in vivo experiment due to methodological differences between in vitro and in vivo experiment. Therefore, further studies using our animal model are warranted on the use of MitoQ and mitochondrial dynamics, including considerations of dose and treatment duration.

Analysis of NOX revealed that the D-CON group exhibited increased p47 phox, gp91 phox, and p22 phox expression in the hippocampus compared with the Y-CON group. Further, IHC analysis revealed increased p22 phox immunoreactivity in the cerebral cortex and hippocampal region CA3. These results are consistent with a previous study reporting that D-gal increased NOX expression in a D-gal-induced aging model [40]. On the other hand, TE downregulated the expression of NOX2 subunits p47 phox, gp91 phox, and p22 phox. In other words, TE effectively inhibited NOX expression, which can induce ROS. This finding concurs with a study in which exercise training reduced NOX activity and restored skeletal muscle mass in rats with heart failure [41] and a study in which TE downregulated NOX expression in the aorta of a high-fat diet-induced obesity model [42]. Notably, MitoQ, which affected mitochondrial dynamics, exerted a strong positive effect on NOX activity. MitoQ treatment alone decreased NOX activity, similar to TE. Unsurprisingly, the same results were also observed for the TE and MitoQ combination treatment. A relevant study reported the protective effect of MitoQ against oxidative stress on mtDNA via activation of Nrf2/ARE signaling, which is a decisive factor in protecting cells from oxidative stress [43]. Therefore, our findings suggest that exercise and MitoQ might ameliorate aging-induced oxidative stress through NOX-related signal pathways [44,45].

To investigate the effects of D-gal-induced aging on neuroinflammation, we also analyzed expression patterns of the astrocyte-specific marker GFAP, proinflammatory factors COX-2 and TNF-α, and the antioxidant enzymes SOD-2 and catalase in the cerebral cortex and hippocampal DG. We observed morphological changes with anti-GFAP staining; cell bodies and dendrites were broader and more strongly stained in the D-CON group compared to the Y-CON group. This suggests that aging is closely related to gliosis, in which activation of astrocytes in the cerebral cortex and hippocampal DG leads to increased neuroinflammation. TE and MitoQ downregulated GFAP expression, thereby decreasing neuroinflammation. These results are consistent with the finding that D-gal increased GFAP expression in brain tissue [46], that astrocyte activity caused innate and adaptive immune responses [47], and that astrocytes in injured brain tissue exhibited elevated GFAP expression [48]. In this study, we confirmed previous studies’ findings that aging is closely associated with neuroinflammation. However, we also found that TE and MitoQ effectively mitigated this neuroinflammation. Specifically, MitoQ treatment demonstrated a clearer effect in the cerebral cortex than in the hippocampal DG. Proinflammatory factors COX-2 and TNF-α demonstrated elevated expression after D-gal treatment, but this was effectively alleviated by TE and MitoQ. The mechanisms underlying the anti-inflammatory effects of exercise have not been clearly elucidated. However, considering the hypothesis that fat reduction is involved [49], increasing physical activity levels will actively utilize the energy stored in adipocytes, and the resulting decrease in body fat mass could be connected to decreased proinflammatory factors secreted by adipocytes. In addition, TE and MitoQ combination treatment increased the expression of antioxidant enzymes SOD-2 and catalase. This concurs with other studies reporting increased antioxidant enzyme levels in aging rats following exercise [50] or MitoQ treatment [51]. Thus, our study findings indicate that exercise and MitoQ helped reduce inflammation and promoted antioxidant function in brain tissue.

We employed the well-studied Morris water maze and passive avoidance tests to investigate whether TE and MitoQ could suppress the commonly observed cognitive decline in aging, including learning and memory. The D-CON group took significantly longer (escape latency time) and traveled further (escape distance) than the Y-CON group to find the escape platform in the water maze test. This demonstrates that D-gal-induced aging reduced learning and memory ability in the experimental animals. On the other hand, TE reduced the escape latency time, demonstrating that TE effectively restored spatial learning and memory. The escape distance also increased slightly, but this was judged to be due to the increased movement speed of the animals after TE. Similarly, in the passive avoidance task, the D-CON group took less time to enter the dark rear chamber from the bright front chamber (retention latency time) compared with the Y-CON group, indicating impaired memory. The results from the water maze and passive avoidance tests suggest that TE can effectively inhibit cognitive decline, including learning and memory that can occur during aging. A previous study reported that MitoQ administration for 5 months improved the impairment of spatial memory retention in a transgenic model of Alzheimer’s disease [52]. Unlike this long-term intake, our results showed that a relatively shorter period of MitoQ treatment failed to improve existing age-induced cognitive decline. Given that MitoQ treatment has positive effects on inflammation, NOX, and antioxidant enzymes in our study, a longer period of MitoQ may be required to prevent aging-related cognitive dysfunction.

The hippocampus is generally considered to be the most important brain region for learning and memory [53]. Considering that animals with hippocampal injury exhibit reduced learning and memory [54], the cognitive-enhancing and memory-improving effects of TE can be understood in terms of increased neuroplasticity and neurogenesis and decreased oxidative stress and inflammation. However, despite reducing oxidative stress and inflammation, MitoQ did not show a clear effect on learning and memory. These results are consistent with a previous study with aged mice (19 months old), in which exercise, but not a diet containing antioxidants, improved memory function [6]. We surmise a possible explanation as to why the MitoQ did not improve age-induced cognitive dysfunction was because, unlike TE, MitoQ does not improve the circulatory function (such as increasing the supply of oxygen and nutrients and removal of waste products and CO_2_ via muscle contraction), and, in particular, has no effect on energy metabolism in the brain (such as increasing levels of neurotransmitters or neurotrophic factors, such as nerve growth factor or brain-derived neurotrophic factor) [55,56,57]. However, this remains only a hypothesis because these factors were not examined in the present study. Nevertheless, our results suggest that MitoQ did not affect neuroplasticity and neurogenesis required to improve actual cognitive function.

## 5. Conclusions

In this study, we confirmed that D-gal-induced aging was associated with cognitive impairment affecting learning and memory due to an imbalance between mitochondrial fusion and fission, increased inflammation, reduced antioxidant enzyme levels, and NOX activation. However, TE ameliorated these pathological effects and prevented cognitive decline related to brain aging. Treatment with MitoQ showed positive effects on inflammation, antioxidant enzymes, and NOX expression but did not improve mitochondrial dynamics or cognitive ability. In turn, in contrast to our hypothesis, the combination of TE and MitoQ did not induce any synergistic or additive effects on the outcome measures we investigated in this study. However, our findings demonstrate that treatment with mitochondria-targeted antioxidants, such as MitoQ, can produce some positive effects. In particular, physical activity, such as TE, more effectively prevents cognitive impairment in aging, including learning and memory. Therefore, we propose that the importance of regular physical activity must be emphasized as an effective non-pharmacological treatment for improving and preventing cognitive decline for older adults.

## Data Availability

The data presented in this study are available on request from thecorresponding author.
